# Demographic and disease-related factors impact bone turnover and vitamin D in children with hemato-oncological diseases

**DOI:** 10.1093/jbmrpl/ziae017

**Published:** 2024-02-24

**Authors:** Natalja Jackmann, Jan Gustafsson, Pauliina Utriainen, Per Magnusson, Arja Harila, Diana Atanasova, Carina Rinaldo, Per Frisk, Outi Mäkitie

**Affiliations:** Department of Women’s and Children’s Health, Uppsala University and University Children’s Hospital, Uppsala 75185, Sweden; Department of Women’s and Children’s Health, Uppsala University and University Children’s Hospital, Uppsala 75185, Sweden; Children’s Hospital, Pediatric Research Center, University of Helsinki and Helsinki University Hospital, Helsinki 00014, Finland; Department of Clinical Chemistry, and Department of Biomedical and Clinical Sciences, Linköping University, Linköping 58183, Sweden; Department of Women’s and Children’s Health, Uppsala University and University Children’s Hospital, Uppsala 75185, Sweden; Department of Clinical Chemistry, and Department of Biomedical and Clinical Sciences, Linköping University, Linköping 58183, Sweden; Department of Women's and Children's Health, Karolinska Institute, Stockholm 17177, Sweden; Department of Women’s and Children’s Health, Uppsala University and University Children’s Hospital, Uppsala 75185, Sweden; Children’s Hospital, Pediatric Research Center, University of Helsinki and Helsinki University Hospital, Helsinki 00014, Finland; Department of Molecular Medicine and Surgery, Karolinska Institute, and Clinical Genetics, Karolinska University Hospital, Stockholm 17177, Sweden

**Keywords:** biochemical markers of bone turnover< bone modeling and remodeling, tumor-induced bone disease < cancer, other< diseases and disorders of/related to bone

## Abstract

Children with hemato-oncological diseases may have significant skeletal morbidity, not only during and after treatment but also at the time of diagnosis before cancer treatment. This study was designed to evaluate the vitamin D status and circulating bone metabolic markers and their determinants in children at the time of diagnostic evaluation for hemato-oncological disease.

This cross-sectional study included 165 children (91 males, median age 6.9 yr range 0.2–17.7 yr). Of them, 76 patients were diagnosed with extracranial or intracranial solid tumors, 83 with leukemia, and 6 with bone marrow failure. Bone metabolism was assessed by measuring serum 25OHD, PTH, bone alkaline phosphatase, intact N-terminal propeptide of type I procollagen, and C-terminal cross-linked telopeptide of type I collagen.

Vitamin D deficiency was found in 30.9% of children. Lower 25OHD levels were associated with older age, lack of vitamin D supplementation, season outside summer, and a country of parental origin located between latitudes −45° and 45°. Children diagnosed with leukemia had lower levels of markers of bone formation and bone resorption than those who had solid tumors or bone marrow failure.

In conclusion, vitamin D deficiency was observed in one-third of children with newly diagnosed cancer. Bone turnover markers were decreased in children with leukemia, possibly because of the suppression of osteoblasts and osteoclasts by leukemic cells. The identification of patients with suboptimal vitamin D status and compromised bone remodeling at cancer diagnosis may aid in the development of supportive treatment to reduce the adverse effects of cancer and its treatment.

## Graphical Abstract

**Graphical Abstract:**
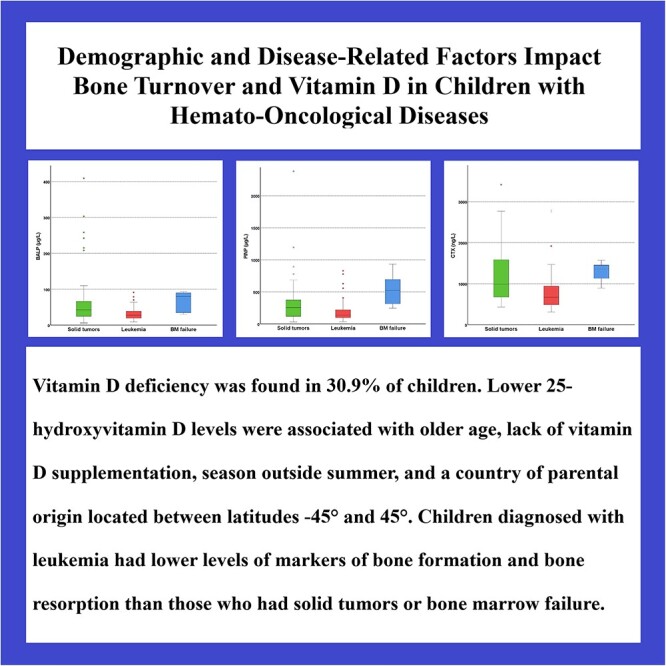

## Introduction

Previously published studies have indicated that children with hemato-oncological diseases may suffer from skeletal morbidity at diagnosis, as well as during and after treatment.^1^ Most studies have focused on children with acute lymphoblastic leukemia (ALL), in whom low bone mineral density (BMD) correlates with the presence of vertebral fractures within 30 d of chemotherapy initiation.^2^ In addition, it predicts vertebral and non-vertebral fractures in the subsequent years.^3^ Studies on skeletal morbidity in children with other types of cancer such as chronic myeloid leukemia,^4^ osteosarcoma and Ewing sarcoma,^5^ neuroblastoma,^6^ and lymphoma^7^ are scarce, reporting that skeletal complications are common and should be considered in the long-term follow-up of these patients.

The cause of skeletal morbidity is multifactorial and involves several disease- and treatment-related factors.^1^ At the time of diagnosis, these include the direct effects of malignant cells on the skeleton as well as the effects of inflammation, immobility, and compromised nutrition.^1^^,^^8^ During cancer treatment, skeletal health may be further influenced by several factors including chemotherapy, glucocorticoids, radiation, low physical activity, and endocrine deficits.^1^^,^^9^ These effects have long-term consequences, and children with hemato-oncological diseases are still at risk of skeletal morbidity several decades after cessation of treatment.^10^

To maintain a healthy skeleton, damaged or old bones must be replaced. Bone remodeling is a constant process throughout life. The initiating event for bone remodeling is bone resorption by osteoclasts followed by bone formation by osteoblasts.^11^ The bone metabolism is influenced by trace elements such as iron, calcium, phosphorus, and magnesium^12^ and vitamins such as vitamin D,^13^ vitamin A,^14^ and vitamin K^15^ and promoted by growth hormone.^16^ A range of biochemical bone turnover markers (BTM) is available for the evaluation of bone formation and resorption, providing a dynamic picture of bone homeostasis. The bone formation markers bone alkaline phosphatase (BALP), intact N-terminal propeptide of type I procollagen (PINP), and osteocalcin represent the activity of osteoblasts, whereas bone resorption markers, such as C-terminal cross-linked telopeptide of type I collagen (CTX) and tartrate-resistant acid phosphatase isoform 5b, reflect the activity of osteoclasts.^17^^,^^18^ However, only a few studies have explored BTM in children with hemato-oncological diseases.^8^^,^^19–22^ Investigating these markers is important for understanding the pathophysiology of skeletal complications in children with hemato-oncological diseases.

Our previous studies, performed in historical cohorts, indicated a high prevalence of vitamin D deficiency in Swedish children diagnosed with cancer.^23^^,^^24^ These observations prompted us to conduct a larger prospective study investigating vitamin D status and BTM in children with hemato-oncological diseases. The aim of this study was to investigate the associations between demographic and disease-related factors and vitamin D status and BTM in children with hemato-oncological diseases at the time of diagnosis.

## Materials and methods

### Patients and study design

**Figure 1:**
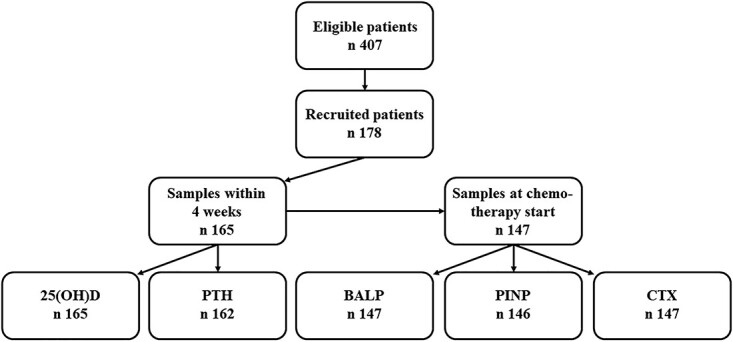
Flow diagram showing the number of eligible and recruited patients and number of available samples.

Patients were recruited from the University Children’s Hospital, Uppsala, Sweden (between July 2015 and November 2019) and the Astrid Lindgren Children’s Hospital, Karolinska University Hospital, Stockholm, Sweden (between April 2016 and November 2017). We invited all children aged 0–17 yr who were diagnosed with cancer or bone marrow failure (BM failure) syndrome during the recruitment period. The considered sample size was calculated as at least 10 observations for each category which was included in the linear regression analyses plus additional 10% for missing values. Of the 407 children diagnosed with cancer or BM failure syndrome during the study period, 178 (44%) were recruited. Thirteen children did not provide blood samples within 4 wk after the start of chemotherapy and were therefore excluded from the analyses (Figure 1). A total of 165 children (91 males) consented to participate and provided the samples. Written informed consent was obtained from children and/or their guardians. The Regional Ethical Review Board of Uppsala, Sweden, approved the study (2014/511).

The aims of the study were first (1) to establish the prevalence of vitamin D deficiency in children with hemato-oncological diseases and (2) to determine which independent variables are associated with 25-hydroxyvitamin D (25OHD) levels. The evaluated variables included age (in years) as a continuous variable and sex, body mass index (BMI) Z-score (5 groups: with BMI Z-score below −2, −2 to −1, −1 to 1, 1 to 2, and above 2), vitamin D supplementation (supplemented vs non-supplemented), season (summer, fall, winter, spring), skin type (type I to type VI), the country of parental origin (north of latitude 45° vs latitude −45° to 45°), and diagnosis (solid tumors, leukemia, and BM failure) as categorical variables (Table 1). Second, we aimed (1) to determine the levels of BTM in children with hemato-oncological diseases and to evaluate whether they are associated with different diagnoses, (2) to evaluate whether the 25OHD and BTM levels are associated with a history of previous bone fractures, and (3) to explore associations between 25OHD and parathyroid hormone (PTH) or BTM.

**Table 1:** Characteristics of the study population (*N* = 165). Distribution of 25OHD according to sex, age, BMI Z-score, vitamin D supplementation, season, skin type, country of parental origin, and diagnosis.

Groups	*N*	Mean 25OHD nmol/L (SD)	95% CI	Deficiency (%)
All patients	165	64.6 (24.5)	60.8–68.4	30.9
Sex Male Female	91 74	64.5 (24.7) 64.7 (24.3)	59.3–69.6 59.1–70.4	31.9 29.7
Age (yr) at diagnosis 0.0–2.0 2.1–6.0 6.1–10.0 10.1–15.0 15.1–18.0	17 54 35 40 19	74.8 (27.1) 69.1 (24.7) 64.7 (16.3) 55.0 (24.5) 62.6 (28.9)	60.9–88.8 62.3–75.8 59.1–70.3 47.1–62.9 48.7–76.5	11.8 27.8 17.1 47.5 47.4
BMI Z-score <−2 −2 to −1 −1 to 1 1 to 2 > 2	11 20 105 21 8	62.5 (30.0) 57.3 (19.6) 67.4 (23.2) 59.9 (29.0) 61.8 (30.5)	42.3–82.7 48.1–66.4 62.8–71.9 46.6–73.1 36.2–87.4	36.4 35.0 24.8 42.8 62.5
Vitamin D supplementation Yes No Missing	46 109 10	75.0 (21.1) 60.6 (24.8)	68.7–81.2 55.765.2	13.1 36.7
Season Summer Fall Winter Spring	35 40 40 50	76.2 (26.4) 63.9 (25.5) 66.2 (22.3) 55.8 (20.6)	67.1–85.3 55.7–72.1 59.0–73.3 49.9–61.7	0.0 27.5 22.5 48.0
Skin type^a^ Type I Type II Type III Type IV Type V Missing	4 73 54 28 5 1	53.8 (21.1) 71.3 (23.9) 59.5 (25.2) 58.1 (22.7) 71.7 (17.3)	20.1–87.5 65.7–76.9 52.6–66.3 49.3–66.9 50.1–93.3	50.0 19.1 40.8 42.8 0.0
Country of parental origin North of latitude 45° Latitude −45° to 45° Missing	104 56 5	70.0 (24.3) 54.1 (21.1)	65.3–74.8 48.4–59.8	23.1 44.6
Diagnosis Solid tumors Leukemia BM failure	76 83 6	61.8 (22.1) 67.7 (26.9) 56.6 (9.2)	56.7–66.9 61.8–73.6 46.8–66.3	31.6 31.3 16.7

a Refers to grading according to the Fitzpatrick scale, in which type I signifies the lightest and type VI signifies the darkest skin complexion.

Clinical data were collected from the medical records, including history of bone fractures, body weight measurements in kilograms, and body height measurements in centimeters, rounded to the tenths. The BMI Z-score was calculated according to WHO Child Growth Standards.^25^ Questionnaires were administered to determine skin types I to VI according to the Fitzpatrick skin scale,^26^ country of parental origin, and vitamin D supplementation. The 4 seasons were summer (June–August), fall (September–November), winter (December–February), and spring (March–May).

### Biochemical measurements

Blood samples were collected from all study participants at the time of diagnosis, usually in the morning, and serum aliquots were stored at −75°C until assayed with reagents from the same batch in September 2022. Serum 25OHD, intact PTH, and BALP levels were analyzed at the Department of Clinical Chemistry (Swedac accredited no. 1342), Linköping University Hospital, Sweden. Serum 25OHD levels were measured using a LIAISON® XL analyzer with a 25OHD total chemiluminescence immunoassay (DiaSorin), which demonstrates 100% cross-reactivity for 25OHD_2_ and 25OHD_3_. The 25OHD method has an analytical range of 10–375 nmol/L and a total coefficient of variation (CV) of ≤8%. Intact PTH was determined with the Elecsys electrochemiluminescence immunoassay on a Roche Cobas e601 platform (Roche Diagnostics Scandinavia AB), with an assay performance of analytical range 0.13–530 pmol/L and total CV of ≤7%. Serum BALP was measured using the BAP Ostase® chemiluminescence immunoassay on a LIAISON® XL analyzer (DiaSorin, Stillwater) with an analytical range of 1.5–120 μg/L and intra- and inter-assay CVs of 7.0%.

Intact PINP was assessed using the UniQ radioimmunoassay (Aidian Oy), with an analytical range of 5–250 μg/L, intra-assay CV of <5%, and inter-assay CV of <6%. CTX was assessed using the serum CrossLaps® enzyme-linked immunosorbent assay (Immunodiagnostic Systems Holdings PLC), with an analytical range of 20–3380 ng/L, intra-assay CV of <6%, and inter-assay CV of <10%.

The levels of 25OHD < 50 nmol/L and < 25 nmol/L were defined as vitamin-D deficiency and severe deficiency, respectively. The level of 25OHD ≥ 50 nmol/L was defined as vitamin D sufficiency.^27^ The reference interval for PTH in the laboratory was 1.6–6.9 pmol/L. The reference intervals used for BALP, intact PINP, and CTX were those reported by Ladang et al.,^28^ Morovat et al.,^29^ and Rauchenzauer et al.,^30^ respectively.

Serum calcium and albumin levels were determined using standard methods and collected from medical records. Albumin-corrected serum calcium [Ca(Alb) in mmol/L] was calculated according to the formula: [actual serum calcium +0.02 × (42-albumin in g/L)].^31^

### Statistical analyses

Statistical analyses were performed using SPSS version 28 (IBM Corp.). A *P*-value <.05 was regarded statistically significant.

Parameters with a skewed distribution (PTH, BALP, PINP, and CTX) are presented as medians with interquartile ranges (IQR, 25–75 percentiles); otherwise, they are presented as means ± SD and 95%CI. Linear regression was used to explore the effects of the study variables on 25OHD levels.

Spearman’s correlation coefficient (r) was calculated to test the association between 25OHD, PTH, Ca(Alb), and 25OHD, BALP, PINP, and CTX. The Kruskal–Wallis test with Bonferroni adjustment for multiple testing was used to compare BTM between the 3 diagnostic groups: children with solid tumors (including those with extra- and intracranial solid tumors), children with leukemia, and children with BM failure. Mann–Whitney test was used to compare PTH between children with vitamin D deficiency and with vitamin D sufficiency and to compare 25OHD between children who had previously experienced bone fractures and those who had not.

## Results

The study population included 165 children (91 male patients, 55.2%), median age 6.9 yr (range, 0.2–17.7). Of them, 62 (37.6%) participants had extracranial solid tumors [soft tissue sarcoma (15), non-Hodgkin lymphoma (10), Hodgkin lymphoma (11), osteosarcoma (8), nephroblastoma and other nonepithelial renal tumors (7), neuroblastoma and ganglioneuroblastoma (3), Ewing sarcoma (2), germ cell tumor (3), hepatic tumor (2), retinoblastoma (1)]; 14 (8.5%) participants had intracranial solid tumors [medulloblastoma (7), atypical teratoid-rhabdoid tumor (3), germ cell tumor (2), Ewing sarcoma (1), pilocytic astrocytoma (1)]; 83 (50.3%) participants had leukemia [ALL (62), acute myeloid leukemia (17), chronic myeloid leukemia (3), juvenile myelomonocytic leukemia (1)]; and 6 (3.6%) participants had BM failure [aplastic anemia (4), Fanconi anemia (1), myelodysplastic syndrome (1)]. Patient characteristics are presented in Table 1.

In this study population, 26 children had experienced bone fractures previously: 5 in the last 3 mo, 1 in the last 3–12 mo, and 20 more than 12 mo before diagnosis. One hundred thirty-two patients had not experienced bone fractures previously, no data about bone fractures were available for 7 children.

Altogether, 89% of children provided samples before (130 children, 79%) or 1 d after (17 children, 10%) the initiation of chemotherapy. In 18 children (11%), the samples were obtained between days 2 and 22 after the initiation of chemotherapy.

### Questionnaires

Skin type I (unexposed skin is bright white, always burns in response to UV (ultraviolet light), never tans) has been reported in 4, skin type II (unexposed skin is white, burns easily in response to UV, tans minimally) in 73, skin type III (unexposed skin is fair, burns moderately, has average tanning ability) in 54, skin type IV (unexposed skin is light brown, burns minimally, tans easily) in 28, and skin type V (unexposed skin is brown, never burns, tans easily, and substantially) in 5 children. None of the patients reported skin type VI (unexposed skin is black, never burns, tans readily, and profusely). No response was obtained from one patient. Regarding vitamin D supplementation at the time of the study, 46 children (28%) received vitamin D at least 1–3 times per week, whereas 109 children (66%) did not take any vitamin D supplements. No response was obtained from 10 children. The country of parental origin (at least one parent) was between latitudes −45° and 45° for 56 children (34%) and north of latitude 45° (including Sweden located at latitudes 55°–69°) for 104 children (63%). No information was available for 5 children.

### 25OHD status and PTH

For the whole cohort, the mean value of 25OHD was 64.6 ± 24.5 nmol/L. Vitamin D deficiency (25OHD < 50 nmol/L) was found in 30.9% of children (4.2% had severe vitamin D deficiency with 25OHD < 25 nmol/L).

We used linear regression to evaluate whether the independent variables were associated with levels of 25OHD. In unadjusted analyses, a negative association was observed between age and levels of 25OHD (B = −1.0, *P* = .004), and vitamin D supplementation was positively associated with 25OHD (B = 14.3, *P* < .001). We observed seasonal variation in 25OHD levels between summer and autumn (B = −12.2, *P* = .02) and between summer and spring (B = −20.3, *P* < .001); summer was associated with higher levels. Furthermore, we investigated whether darker skin had an influence on vitamin D status. As skin type I was only reported in 4 children, we used skin type II as the reference group. The skin type V group included only 5 patients, 3 of whom had vitamin D supplementation. We found that skin type III (B = −11.8, *P* = .007) and skin type IV (B = −13.1, *P* = .01), but not skin type I or V, were associated with lower levels of 25OHD than those of the reference group (skin type II). In line with this, children with one or both parents originating from a country mainly located between latitudes −45° and 45° had significantly lower 25OHD levels when compared with children with parents from countries located north of latitude 45° (B = −15.9, *P* < .001). None of the participants had parents from countries located south of latitude −45°. There were no significant differences in the 25OHD levels between the sexes nor between the different diagnostic groups or groups with different BMI Z-score. After adjusting for all variables with *P* < .1, age (B = −0.85, *P* = .025), vitamin D supplementation (B = 11.0, *P* = .007), season (fall, winter, and spring compared with summer, B = −12.5, *P* = .01; B = −11.6, *P* = .02; and B = −19.3, *P* < .001, respectively), and parental origin (B = −13.1, *P* = .002), but neither skin type nor BMI Z-score remained significant predictors of 25OHD levels (Table 2).

**Table 2:** Factors influencing levels of 25OHD among 165 children with hemato-oncological diseases.

**Linear regression**	**Unadjusted analyses**			**Adjusted analyses**		
	B	R^2^	*P*	B	Beta	*P*
All patients (165)						
Sex Male (Ref.) Female	0.2	0.000	.9			
Age (yr)	−1.0	0.050	.004^a^	−0.85	−0.176	.025^a^
BMI Z-score <−2 −2 to −1 −1 to 1 (Ref.) 1 to 2 > 2	−4.8 −10.1 −7.4 −5.5	0.025	.5 .09 .2 .5	0.6 −7.8 −9.0 −6.7	0.006 −0.105 −0.126 −0.057	.9 .1 .9 .4
Vitamin D supplement No (Ref.) Yes	14.3	0.071	<.001^a^	11.0	0.205	.007^a^
Season Summer (Ref.) Fall Winter Spring	−12.2 −9.9 −20.3	0.088	.02^a^ .06 <.001^a^	−12.5 −11.6 −19.3	−0.220 −0.203 −0.358	.01^a^ .02^a^ <.001^a^
Skin type^b^ Type I Type II (Ref.) Type III Type IV Type V	−17.5 −11.8 −13.1 0.4	0.067	.1 .007^a^ .01^a^ .9	−15.4 −6.4 −2.8 3.6	−0.100 −0.124 −0.042 0.026	.1 .1 .6 .7
Country of parental origin North of latitude 45° (Ref.) Latitude −45° to 45°	−15.9	0.097	<.001^a^	−13.1	−0.253	.003^a^
Diagnosis Solid tumors (Ref.) Leukemia BM failure	5.8 −5.2	0.018	.1 .6			
R^2^	0.391			0.319		

a Regression is significant.

b Refers to grading according to the Fitzpatrick scale, in which type I signifies the lightest and type VI signifies the darkest skin complexion.

Using Mann–Whitney test, we could not identify any differences in 25OHD levels between children who had previously experienced bone fractures as compared with those who had not.

**Figure 2:**
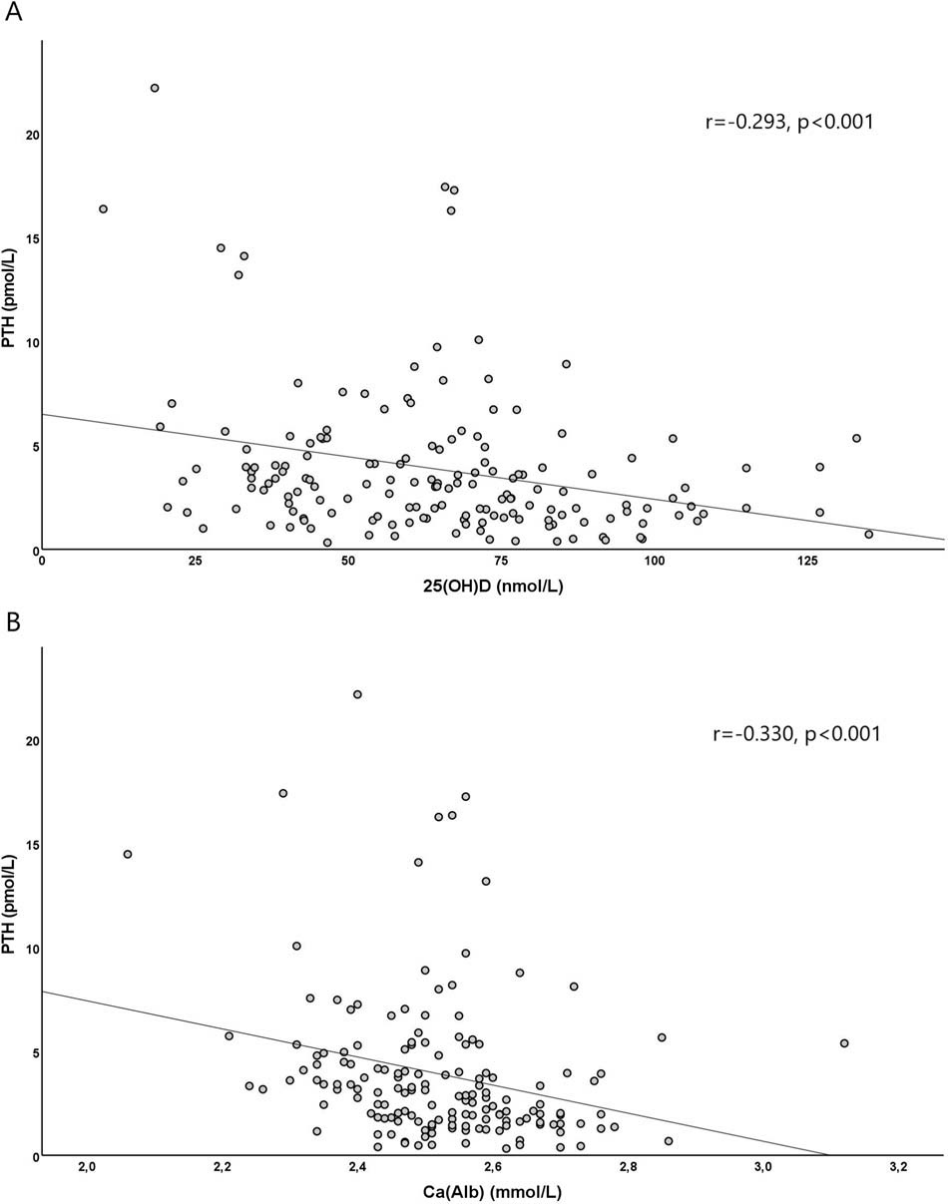
Correlation between PTH and 25OHD (A) and PTH and Ca(Alb) (B) in 165 children with hemato-oncological diseases.

Serum PTH levels were available for 162 patients (98.2%). The median PTH value was 2.97 pmol/L (IQR = 1.62–4.79 pmol/L) and the values were normal in 64.2%: 23.5% of the children displayed levels below and 12.3% above the reference interval (1.6–6.9 pmol/L). Inverse correlations were observed between PTH and 25OHD (r = −0.293, *P* < .001) and PTH and Ca(Alb) (r = −0.330, *P* < .001) (Figure 2). PTH level was higher in children with vitamin D deficiency as compared with those with vitamin D sufficiency (*P* = .015).

### BALP, PINP, and CTX levels

**Figure 3:**
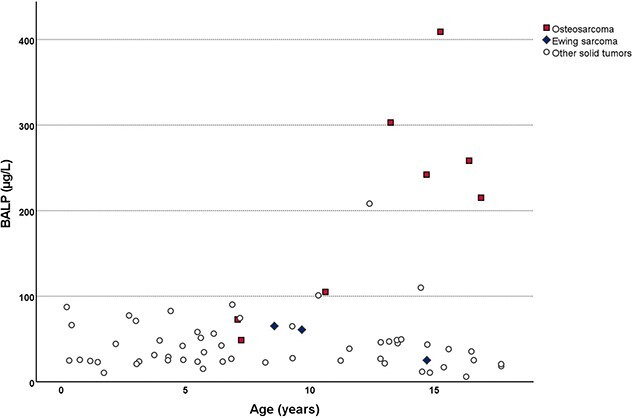
Distribution of BALP in 8 children with osteosarcoma, 3 children with Ewing sarcoma and in 55 children with other solid tumors.

BTM reflects the bone metabolism and may change rapidly with the onset of chemotherapy. We found that PINP levels were lower in children with leukemia who had blood samples drawn after the start of the chemotherapy as compared with children who had the blood samples drawn at the time of chemotherapy initiation (*P* = .04). To minimize bias, further analyses included only the 147 patients who had blood samples available at the time of chemotherapy initiation (66 with solid tumors, 75 with leukemia, and 6 with BM failure). Serum BALP measurements were available for 147 patients. The median BALP value was 31.2 μg/L (IQR = 21.6–50.2 μg/L), 75.5% had a value within the normal reference range, 20.4% of the children displayed values below and 4.1% above the reference interval. Five of 6 children with very high BALP values (208–409 μg/L, all above the 96th percentile) were diagnosed with osteosarcoma (Figure 3). Three additional children with osteosarcoma displayed normal BALP levels.

Serum PINP and CTX levels were available for 146 and 147 patients, respectively. The median PINP value was 184.7 μg/L (IQR = 108.7–334.5 μg/L) and only 47.3% had a value within the normal reference range. More than half (51.4%) of the children displayed levels below and 1.4% displayed levels above the reference interval. The median CTX level was 823.5 ng/L (IQR = 568.5–1240.5 ng/L). The values were mostly normal (78.9 %); however, 20.4% of the children displayed levels below and 0.7% above the reference interval (Table 3).

**Table 3:** Biochemical bone turnover markers in children with hemato-oncological diseases at time of diagnosis in comparison with age- and sex-adjusted reference intervals.

**Diagnosis**	**BALP** ^**28**^			**PINP** ^**29**^			**CTX** ^**30**^		
	Low *N* (%)	Norm *N* (%)	High *N* (%)	Low *N* (%)	Norm *N* (%)	High *N* (%)	Low *N* (%)	Norm *N* (%)	High *N* (%)
Solid tumors	12 (15.8)	58 (76.3)	6 (7.9)	28 (37.3)	45 (60)	2 (2.7)	6 (7.9)	70 (92.1)	0 (0)
Leukemia	22 (26.5)	61 (73.5)	0 (0)	60 (72.3)	23 (27.7)	0 (0)	27 (32.5)	55 (66.3)	1 (1.2)
BM failure	0 (0)	6 (100)	0 (0)	0 (0)	6 (100)	0 (0)	0 (0)	6 (100)	0 (0)
All	34 (20.6)	125 (75.8)	6 (3.6)	88 (53.7)	74 (45.1)	2 (1.2)	33 (20)	131 (79.4)	1 (0.6)

In the whole study population, all BTM were within the reference ranges in only 41.5% of children. Overall, 86 children (58.5%) displayed at least one BTM (BALP, PINP, or CTX) below the reference interval. This proportion was higher in children with leukemia (83.3%) than in those with solid tumors (37.9%). All 6 children with BM failure displayed normal BALP, PINP, and CTX values according to the reference intervals.^28–30^

**Figure 4:**
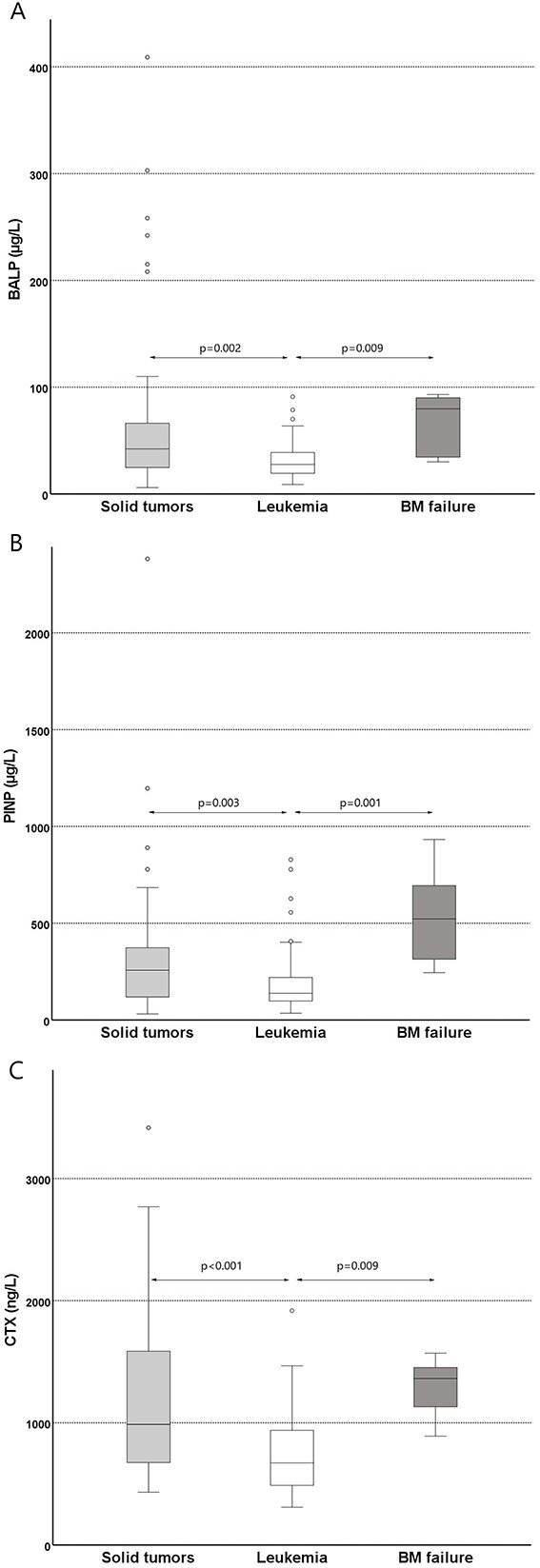
Distribution of serum (A) BALP, (B) PINP, and (C) CTX in children with solid tumors (66), leukemia (75), and bone marrow failure (6). Statistical analyses were done by Kruskal–Wallis test with Bonferroni adjustment.

A comparison of BTM between the diagnostic groups demonstrated that BALP, PINP, and CTX levels were significantly lower in children with leukemia than in those with solid tumors (*P* = .002, *P* = .003, *P* < .001, respectively) or BM failure (*P* = .009, *P* = .001, *P* = .009, respectively) (Figure 4). Positive correlations were observed between BALP and PINP (r = 0.593, *P* < .001), BALP and CTX (r = 0.385, *P* < .001), and PINP and CTX levels (r = 0.316, *P* < .001). There was no correlation between BTM and 25OHD.

Furthermore, we stratified the data of 147 patients by vitamin D supplementation: 98 patients did not receive vitamin D supplementation (41 with solid tumors, 53 with leukemia, and 4 with BM failure), 41 patients were vitamin D supplemented (22 with solid tumors, 17 with leukemia, and 2 with BM failure). In 8 patients, vitamin D supplementation status was unknown. In the vitamin D non-supplemented group, the comparison of BTM between the diagnostic groups demonstrated that BALP, PINP, and CTX levels were significantly lower in children with leukemia than in those with solid tumors (*P* = .006, *P* = .009, and *P* < .001, respectively) or BM failure (*P* = .035, *P* = .007, *P* = .047, respectively). In contrast, in the vitamin D supplemented group, there were no statistically significant differences in BTM across the diagnostic groups, possibly due to the low number of participants.

We then analyzed whether the children with a history of skeletal fractures had changes in BTM. In this group of 147 children, 23 had experienced bone fractures previously and 118 had not. No data regarding bone fractures were available for 6 children. Using Mann–Whitney test, we showed that children with a history of bone fractures had lower levels of BALP (*P* = .007) and PINP (*P* = .009), meanwhile CTX levels were comparable in children with and without bone fractures. However, children with history of fractures were older (95% CI for mean 9.0–13.0 yr) than children without fractures (95% CI for mean 6.6–8.5 yr, *P* = .004) and this probably contributed to the observed differences in BTM.

## Discussion

Our results demonstrate that one-third of the children with hemato-oncological diseases in Sweden are vitamin D deficient at the time of diagnosis. Furthermore, in two-thirds of the children, at least one BTM was decreased, possibly indicating compromised bone metabolism. Similar results on vitamin D deficiency were obtained in our previously published study on historical cohorts of children with hematological and non-hematological malignancies,^23^^,^^24^ as well as in a study on healthy Swedish children,^32^ implying that children with hemato-oncological diseases and healthy children in Sweden may carry a comparable risk of vitamin D deficiency. Furthermore, previous studies reported that approximately 28%–63% of healthy European children have 25OHD levels <50 nmol/L.^33–38^ The frequently reported factors associated with vitamin D deficiency in pediatric populations are older age, overweight, winter/spring season, dark skin, female sex, parental vitamin D deficiency, lack of vitamin D supplementation, and lifestyle with less outdoor exercise and reduced activity and fitness.^33^^,^^37^^,^^39–43^ The highest prevalence of vitamin D deficiency in children with cancer, 41% (range 21%–62%), was reported among European children, compared with 24% (range 24–25%) among children in the Middle East and 15% (range 0–16%) among children in North America.^44^

The synthesis of vitamin D in the skin is genetically determined: the more melanin in the skin, the lower the amount of previtamin D_3_ that can be synthesized in response to UV radiation.^45^ The skin color of indigenous populations becomes darker from the poles to the equator, and is therefore associated with the ethnic background. In this study, we used 2 tightly connected variables: reported skin type (I–VI according to the Fitzpatrick scale^26^) and country of parental origin. As expected, vitamin D deficiency was more common in children with darker skins. In our study cohort, only a few children had skin types I and V and none had skin type VI. However, children with skin types III and IV had a prevalence of vitamin D deficiency twice as high as that of children with skin type II. These data are in agreement with those of Åkeson et al., who reported that low 25OHD levels are associated with a darker skin type in children in Sweden.^46^ However, in our study, the country of parental origin was a more powerful variable associated with 25OHD levels than skin type. Children of parents from countries located south of latitude 45° had 15.9 nmol/L lower mean for 25OHD than children of parents from countries located north of latitude 45°. This indicates that immigrant populations with dark skin who live in the northern part of the globe have a greater risk of vitamin D deficiency. Parental origin should be considered a possible risk factor for the prevention of vitamin D deficiency. These results are consistent with systematic review and meta-analysis data on the prevalence of vitamin D deficiency in pediatric cancer patients worldwide, reporting that the lower levels of 25OHD were associated with darker skin.^44^ In agreement with previous studies,^32^^,^^38^^,^^44^^,^^47–52^ we showed that the levels of 25OHD were negatively associated with age; consequently, vitamin D deficiency was more common in older children and adolescents. The mechanisms underlying this finding remain unclear. It could be speculated that older children receive less sun exposure because they spend less time outdoors than younger children do. They may also consume less vitamin D in their diet because they eat less fish and vitamin D-fortified products. Older children may also have higher absolute fat mass, which may affect the distribution of vitamin D.

In our study cohort, children with ongoing vitamin D supplementation had a lower risk of vitamin D deficiency, supporting that vitamin D supplementation may effectively prevent vitamin D deficiency. In line with previous studies,^24^^,^^47^ there was seasonal variation in 25OHD concentration, with the lowest level in spring and the highest level in summer.

In line with previous studies,^24^^,^^47^^,^^53^ PTH correlated inversely with 25OHD, consequently 12% of patients displayed secondary hyperparathyroidism. Interestingly, 23% of patients had subnormal levels of PTH. We could not find any specific characteristics in this group of patients regarding diagnosis, 25OHD level, or Ca(Alb). In contrast, another study reported a relationship between PTH and 25OHD in healthy controls, but not in children with cancer.^38^ The explanation for this may be that children with cancer in that study were under cancer treatment which may have influenced the metabolism of PTH.

Previous studies have shown that bone health can be compromised already at the time of cancer diagnosis, especially in children with leukemia. Decreased BMD in children with ALL results in higher fracture rates.^8^^,^^20^^,^^21^^,^^54^ We analyzed a panel of biochemical markers reflecting bone turnover to study the effects of hemato-oncological diseases on bone metabolism at the time of diagnosis. We demonstrated that both markers of bone formation and resorption were lower in children with leukemia than in those with solid tumors or BM failure. These results are in accordance with earlier reports on low levels of bone formation markers in children with ALL, whereas bone resorption markers have been reported to be low or normal.^8^^,^^19–22^ The decrease in BTM in patients with leukemia at diagnosis indicates the effect of the disease itself on bone turnover. The pathogenic mechanisms underlying the effect of leukemic cells on bone cells are unknown. It has been shown in a murine model that leukemic cells may inhibit osteoblastic function by both direct cell contact and secretion of inhibitory molecules.^55^ Interestingly, in line with a previous study,^56^ a large proportion of children with solid tumors displayed low BTM, especially bone formation markers, even though their levels were higher than those in children with leukemia.

The interpretation of BTM in children may be challenging because they reflect not only the bone remodeling process but also skeletal growth. In addition, other factors, such as inflammation, infection, immobilization, malnutrition, and weight loss, may influence BTM by decreasing bone formation, while the effects on bone resorption may be variable.^18^^,^^57^ Such conditions are common in children with leukemia and in children with solid tumors and may play a role in the suppression of bone remodeling. However, children with solid tumors do not have the same type of BM infiltration, which may explain the more suppressed bone turnover observed in children with leukemia. All the children with BM failure had normal levels of BTM.

In our study, we demonstrated that children with a history of bone fractures had lower levels of BALP and PINP as compared with children without fractures. A possible explanation could be that the low levels of BALP and PINP reflected an impaired bone metabolism in these children and a propensity for fractures. Another theory is that those changes in BTM are age-dependent. Consistent with previous studies,^58^ there was no association between lower 25OHD levels and fractures. Whether the measurements of BTM may provide additional benefit in the assessment of risk factors for fractures in this patient group warrants further evaluation.

Notably, 6 children with solid tumors displayed very high levels of BALP. Five of these patients were diagnosed with osteosarcoma, and one with rhabdomyosarcoma with skeletal destruction. All 6 children were older than 12 yr of age. None of them had bone fractures in the last 2 yr and the PTH, PINP, and CTX levels were normal. In contrast, 3 children with osteosarcoma who were younger (7-10 yr of age) displayed normal levels of BALP. These data are in line with previously published results reporting that adolescents and adults with osteosarcoma have higher plasma/serum BALP levels than healthy controls^59^ and patients with other bone tumors but normal levels of CTX.^60^ This could be explained by the fact that pathological osteoblasts in osteosarcoma produce large amounts of BALP.

The risk factors for vitamin D deficiency recognized in the present study (older age, lack of vitamin D supplementation, season outside summer, and country of parental origin located between latitudes −45° and 45°) may differ in other countries, and the importance of each factor must be evaluated separately for each population.

### Clinical implications and further research

Our data on prevalence and risk factors of vitamin D deficiency imply that older age, season outside summer, and ethnicity (country of parental origin between latitudes −45° and 45°) should be considered as risk factors when guidelines for vitamin D supplementation are developed. Furthermore, our results may be used as a basis for interventional studies on vitamin D supplementation. Long-term trials in these patients are needed to evaluate the benefits of vitamin D supplementation. Further studies should be carried out to evaluate whether BTM may be used to predict the risk of fractures and/or osteoporosis, to diagnose skeletal adverse effects, and to monitor treatment of skeletal disease in children with hemato-oncological diagnoses.

### Study limitations and strengths

The major limitation of this study is the lack of healthy controls. Second, not all samples were obtained before the start of chemotherapy, although the proportion of such samples was small. Another limitation is that we were not able to evaluate the dosage of vitamin D supplementation since different products, including off-the-shelf products, were used. Neither sun exposure nor dietary intake could be determined. The major strengths of the study lie in its prospective design and relatively large cohort of children with hemato-oncological diseases. Furthermore, our study provides data on both bone formation and bone resorption markers.

In summary, our study shows that one-third of the children with newly diagnosed hemato-oncological diseases in Sweden are vitamin D deficient. Older children and children in families originating from countries located between latitudes −45° and 45° are at a higher risk of developing vitamin D deficiency. Vitamin D status is positively influenced by vitamin D supplementation. Furthermore, metabolic bone turnover is affected more in children with leukemia than in those with solid tumors. The clinical importance of high BALP levels in adolescents with osteosarcoma requires further evaluation.

These results may be important for the development of strategies to prevent vitamin D deficiency and to understand the risk factors for skeletal morbidity in children with hemato-oncological diseases.

## Data Availability

Data available on request due to privacy/ethical restrictions.
